# Early-fusion hybrid CNN-transformer models for multiclass ovarian tumor ultrasound classification

**DOI:** 10.3389/frai.2025.1679310

**Published:** 2025-10-15

**Authors:** Igor Garcia-Atutxa, José Martínez-Más, Andrés Bueno-Crespo, Francisca Villanueva-Flores

**Affiliations:** ^1^Escuela Politécnica Superior, Universidad Católica de Murcia (UCAM), Murcia, Spain; ^2^Centro de Investigación en Ciencia Aplicada y Tecnología Avanzada (CICATA), Unidad Morelos del Instituto Politécnico Nacional (IPN), Xochitepec, Mexico; ^3^Facultad de Medicina, Universidad Católica de Murcia (UCAM), Murcia, Spain

**Keywords:** ovarian cancer, ultrasound imaging, deep learning, CNN, vision transformer, hybrid model, early diagnosis

## Abstract

Ovarian cancer remains the deadliest gynecologic malignancy, and transvaginal ultrasound (TVS), the first-line test, still suffers from limited specificity and operator dependence. We introduce a learned early-fusion (joint projection) hybrid that couples EfficientNet-B7 (local descriptors) with a Swin Transformer (hierarchical global context) to classify eight ovarian tumor categories from 2D TVS. Using the public, de-identified OTU-2D dataset (*n* = 1,469 images across eight histopathologic classes), we conducted patient-level, stratified 5-fold cross-validation repeated 10×. To address class imbalance while preventing leakage, training used train-only oversampling, ultrasound-aware augmentations, and strong regularization; validation/test folds were never resampled. The hybrid achieved AUC 0.9904, accuracy 92.13%, sensitivity 92.38%, and specificity 98.90%, outperforming single CNN or ViT baselines. A soft ensemble of the top hybrids further improved performance to AUC 0.991, accuracy 93.3%, sensitivity 93.6%, and specificity 99.0%. Beyond discrimination, we provide deployment-oriented evaluation: isotonic calibration yielded reliable probabilities, decision-curve analysis showed net clinical benefit across 5–20% risk thresholds, entropy-based uncertainty supported confidence-based triage, and Grad-CAM highlighted clinically salient regions. All metrics are reported with 95% bootstrap confidence intervals, and the evaluation protocol preserves real-world data distributions. Taken together, this work advances ovarian ultrasound AI from accuracy-only reporting to calibrated, explainable, and uncertainty-aware decision support, offering a reproducible reference framework for multiclass ovarian ultrasound and a clear path toward clinical integration and prospective validation.

## 1 Introduction

Ovarian cancer (OC) remains the most lethal gynecological malignancy worldwide. According to the World Health Organization (WHO), ovarian cancer ranks as the eighth most common cancer among women globally, with approximately 324,603 new cases and 206,956 deaths reported annually, translating to an estimated incidence of 6.7 cases per 100,000 women (Ferlay et al., 2024). Despite significant advances in surgical treatments and targeted therapies, global five-year survival remains below 50%, mainly due to late-stage diagnosis (Dexter et al., 2024; Lheureux et al., 2019). Approximately 66–70% of OC cases are diagnosed at advanced FIGO stages (III–IV), which is tightly associated with poorer five-year survival, where therapeutic efficacy is limited and invasive interventions become necessary, severely impacting patient quality of life and increasing healthcare costs (Hong and Ding, 2025; Siegel et al., 2021; Cortés Morera et al., 2020; Christiansen et al., 2025; Menon et al., 2018).

Currently, B-mode transvaginal ultrasound (TVS) is the primary imaging modality for the initial assessment of suspicious ovarian masses, given its accessibility, low cost, and absence of ionizing radiation (Van Nagell and Hoff, 2013; Sideris et al., 2024). However, despite its relatively high sensitivity, TVS suffers from limited specificity when used in isolation, with real-world specificity varying across practice settings, frequently resulting in diagnostic uncertainty, numerous false positives, and unnecessary invasive procedures (Gareeballah et al., 2025; Almeida et al., 2025; Tsili et al., 2024). Consequently, TVS is often complemented with validated structured criteria, such as IOTA and O-RADS, including the 2022 ACR O-RADS US update, as well as subjective evaluations by expert clinicians, which can lead to observational bias and considerable inter-observer variability (Dexter et al., 2024; Buranaworathitikul et al., 2024; Christiansen et al., 2021), and reproducibility within IOTA frameworks still depends on reader expertise. This underscores the urgent need for automated, objective diagnostic tools that can deliver accurate and reproducible evaluations of ovarian lesions, particularly in clinical settings with limited resources or expertise (Tang et al., 2025; Gao et al., 2022).

Recent advances in artificial intelligence (AI) and deep learning (DL) have revolutionized automated medical image analysis (Mienye et al., 2025). Convolutional neural networks (CNNs) have become the gold standard for complex diagnostic tasks, demonstrating robust performance in various medical imaging domains, from breast and lung tumor identification to classification of brain lesions, due to their exceptional ability to extract local image features (Hong and Ding, 2025; Christiansen et al., 2025; Sideris et al., 2024). In ovarian ultrasound specifically, CNN-based and radiomics-based pipelines have shown promising performance for benign–malignant discrimination and, more recently, multiclass stratification; however, hybrid CNN-Transformer approaches remain uncommon.

Nevertheless, CNN and Vision Transformer (ViT) models individually exhibit notable limitations affecting clinical generalization: CNNs typically underestimate global contextual information, whereas ViTs often struggle to preserve essential fine-grained local features necessary for precise anatomical lesion classification (Khan et al., 2023; Zhang et al., 2024; Kim et al., 2024). Recent studies in other medical domains have demonstrated that hybrid CNN-Transformer architectures significantly enhance performance in complex tasks, such as lung lesion segmentation and breast cancer classification, highlighting their superior capability to manage inherent variability and complexity in medical imaging (Liu et al., 2021; Djoumessi et al., 2025; Mustapha et al., 2025). Yet, the application of these hybrid approaches specifically to ovarian cancer diagnosis from ultrasound images remains underexplored, constituting a critical scientific and clinical gap that limits the potential impact of AI in gynecologic oncology. Only a handful of studies have examined hybrids tailored to ovarian ultrasound compared with other organ systems.

Although recent hybrid CNN-Transformer architectures have been proposed for medical imaging, they typically employ late-fusion strategies, combining CNN and Transformer features at later stages of model processing, thereby limiting early feature interaction and potentially reducing diagnostic accuracy. Unlike previous approaches (Chen et al., 2021; Zhang et al., 2021), our proposed methodology implements a novel learned early-fusion (joint projection). This design explicitly enables early cross-talk between local CNN-extracted features and global Transformer-derived context, a choice motivated by evidence that early interactions can improve generalization over late-fusion baselines. Consequently, this provides substantial methodological innovation with the potential to enhance the performance of multiclass ovarian tumor classification significantly.

In this work, we make several contributions to AI-based ultrasound classification of ovarian tumors. We introduce a learned early-fusion (joint projection) hybrid architecture that couples EfficientNet-B7 with a Swin Transformer, enabling joint optimization and early crosstalk between local and global representations. We conduct a comprehensive multiclass evaluation on the OTU-2D dataset, which mirrors real-world case mix and class imbalance. We adopt class-aware training and reporting, with a specific emphasis on underrepresented categories that are crucial for clinical triage. To support clinical trust and quality assurance, we provide clinician-oriented interpretability via Grad-CAM and rigorously benchmark the proposed model against CNN-only, Transformer-only, and late-fusion baselines, quantifying the added value of early fusion. Beyond single-model performance, we further optimize diagnostic accuracy through a soft ensemble of the top hybrid models. We also move beyond accuracy-only assessments by evaluating probabilistic calibration (via isotonic regression) and clinical utility (via decision curve analysis). Finally, we explicitly characterize predictive uncertainty through an entropy-based analysis, enabling risk-aware automation and escalation policies. We ensure statistical robustness and reproducibility by employing patient-level stratified cross-validation, multiple independent runs with fixed seeds, bootstrap confidence intervals, and formal hypothesis testing.

## 2 Materials and methods

### 2.1 Ethics, data privacy, and security

**Figure 1:**
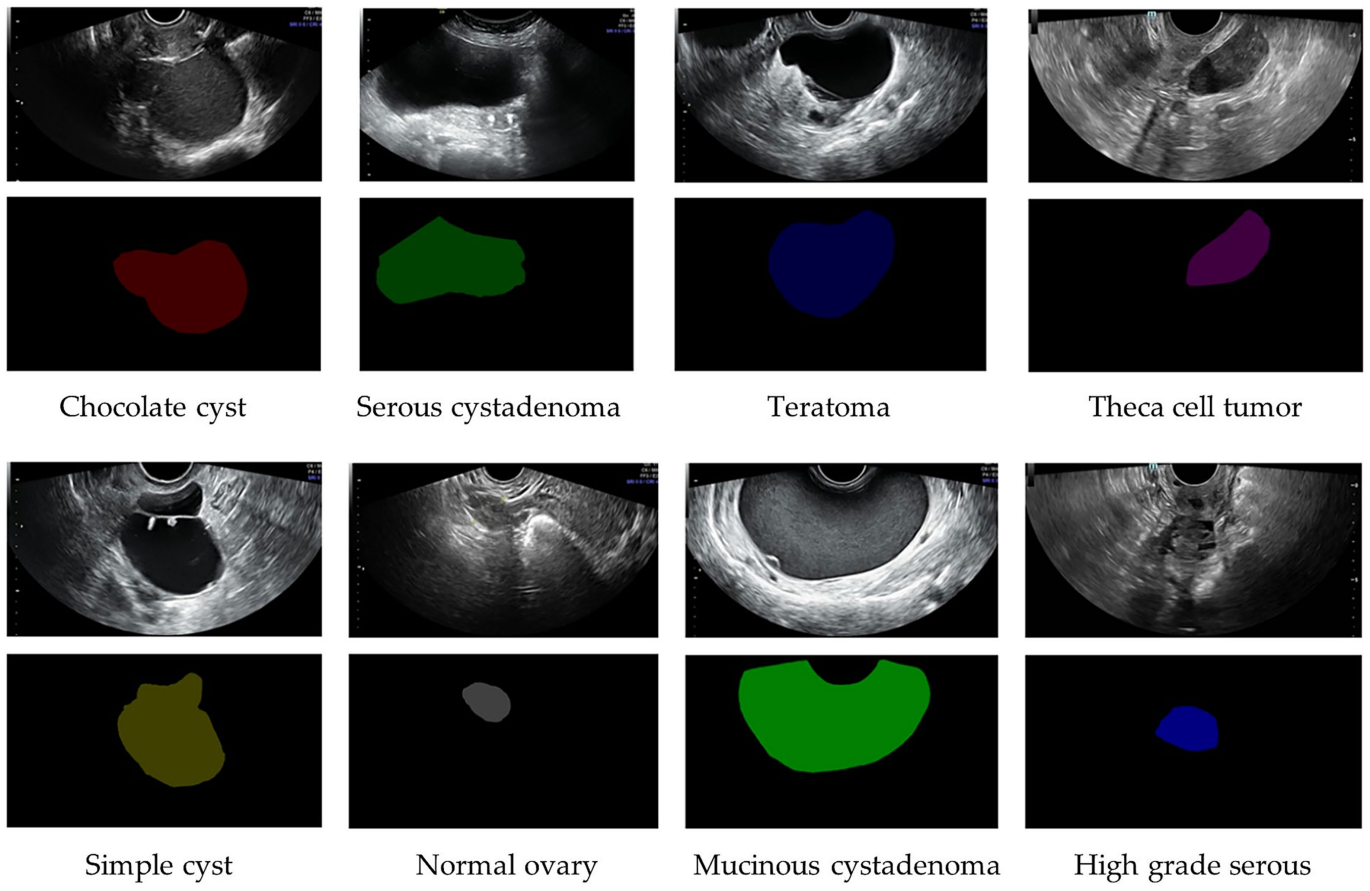
Representative examples of the eight clinical categories included in the OTU-2D dataset (original images and annotations).

The present study utilized the publicly available, de-identified OTU-2D (Ovarian Tumor Ultrasound—2D) dataset, comprising 1,469 two-dimensional B-mode ultrasound images acquired under standardized clinical conditions (Figure 1). Images were retrospectively collected from patients attending Beijing Shijitan Hospital, Capital Medical University, Beijing, China, with all diagnoses histopathologically confirmed by expert pathologists. Experienced gynecologic oncologists systematically classified ovarian lesions into eight clinically relevant diagnostic categories: chocolate cyst (endometrioma), serous cystadenoma, mucinous cystadenoma, teratoma, simple cyst (functional cyst), theca cell tumor, high-grade serous carcinoma, and normal ovary, using the IOTA consensus and O-RADS US (2022) lexicón as specified by the dataset curators. Because OTU-2D is a public resource containing only de-identified images and metadata, this secondary analysis did not require new Institutional Review Board/Ethics Committee approval or patient informed consent. All analyses were performed exclusively on de-identified files; no attempt was made to re-identify individuals or to link records to external sources. Processing followed privacy-by-design safeguards (data minimization; restricted, role-based access; encryption at rest and in transit; and audit logging) and complied with applicable data-protection requirements for secondary research on de-identified data (e.g., GDPR research provisions) (Vara et al., 2023; Garg et al., 2017; Andreotti et al., 2020). A detailed breakdown of the dataset composition per diagnostic class is provided in Table 1.

**Table 1:** Distribution of the OTU-2D dataset used in this study, showing the number of two-dimensional ultrasound images classified into each evaluated clinical category.

Category	Number of images
Chocolate cyst	336
Serous cystadenoma	219
Teratoma	336
Theca cell tumor	88
Simple cyst	66
Normal ovary	267
Mucinous cystadenoma	104
High grade serous	53

### 2.2 Image preprocessing

All ultrasound images were resized to 224 × 224 pixels to standardize the input resolution required by the utilized architectures. Images were converted to numeric tensors and normalized using the RGB channel mean [(0.485, 0.456, 0.406)] and standard deviation [(0.229, 0.224, 0.225)] derived from the ImageNet dataset (Deng et al., 2009), a common practice facilitating convergence and generalization through transfer learning.

In addition, we applied ultrasound-specific data augmentation only to the training data to improve generalization while preserving clinical plausibility, including small in-plane rotations and translations, random crops, additive Rayleigh-distributed speckle noise, brightness/contrast, and time-gain—like intensity perturbations, and acoustic artifacts such as mild shadowing and posterior enhancement.

Due to a significant class imbalance observed in the original dataset, a class rebalancing strategy was required. We opted for random oversampling, which was applied exclusively to the training folds after the stratified patient-level split, thereby increasing the number of examples from minority classes by randomly duplicating images. Validation and test folds were never resampled or augmented to preserve the real-world distribution and prevent information leakage. The rationale behind selecting oversampling rather than focal loss or class-balanced loss was twofold: (Ferlay et al., 2024) oversampling maintains the standard categorical cross-entropy loss function, preserving its interpretability and facilitating training stability, and (Dexter et al., 2024) it directly equalizes class distributions, ensuring adequate feature representation from minority classes without modifying loss dynamics. While focal loss and class-balanced loss address imbalance by modifying loss function gradients, they can complicate training convergence and introduce additional hyperparameter tuning complexity. Thus, oversampling was chosen as a straightforward, effective, and interpretable method to handle class imbalance.

To further mitigate overfitting potentially induced by resampling, oversampling was combined with the ultrasound-specific augmentations and with model-level regularization (dropout and L2 weight decay).

Validation and test sets remained unaltered to ensure an unbiased evaluation of model performance on real-world data distribution.

### 2.3 Deep learning architectures

Five pre-trained architectures were evaluated: three CNN-based (ResNet-152, DenseNet-201, EfficientNetB7) and two Transformer-based (ViT-B16, Swin Transformer). These architectures were selected based on previously demonstrated performance in complex medical imaging tasks: ResNet-152 (He et al., 2015): Utilizes residual connections, enabling adequate gradient flow in deep architectures. DenseNet-201 (Huang et al., 2017): Employs dense connectivity, improving feature reuse and reducing parameter count. EfficientNetB7 (Tan and Le, 2020): Implements automated compound scaling for optimal accuracy and computational efficiency. ViT-B16 (Dosovitskiy et al., 2021): Adapts transformer architecture to visual tasks through global attention mechanisms over image patches. Swin Transformer (Liu et al., 2021): Implements hierarchical attention through shifted-window mechanisms, suitable for capturing complex anatomical structures.

**Figure 2:**
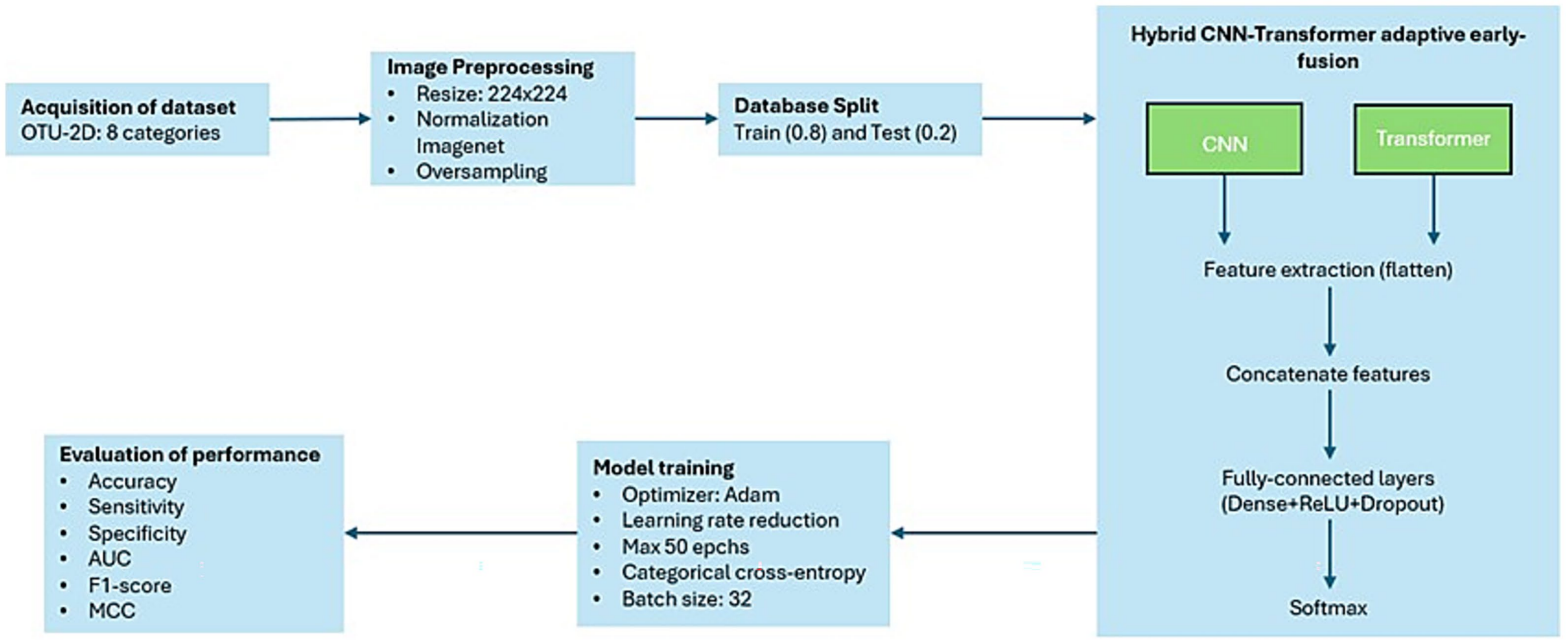
Methodological pipeline. From OTU-2D acquisition and preprocessing to patient-level, stratified 5-fold cross-validation repeated 10×. CNN (local) and ViT (global) branches are combined via a learned early-fusion (joint projection) that enables early co-adaptation of features; training uses train-only oversampling, ultrasound-aware augmentation, dropout, and L2 weight decay. Evaluation includes clinically relevant metrics with 95% CIs, bootstrap resampling, and statistical testing (Shapiro–Wilk, paired *t*/Wilcoxon; ANOVA/Tukey, *α* = 0.01), plus isotonic calibration, decision-curve analysis, entropy-based uncertainty, and Grad-CAM interpretability. Validation/test folds were never resampled or augmented to avoid leakage.

For each proposed hybrid CNN–Transformer model, the selected CNN and Transformer architectures were individually loaded with pre-trained ImageNet weights, and their original classification layers were removed. For the CNN architectures, final convolutional features underwent adaptive average pooling followed by flattening to produce a compact, one-dimensional feature vector. For the Transformer architecture, features were directly obtained as flattened feature vectors from their respective pre-classification layers. Subsequently, CNN-derived local features and Transformer-derived global contextual features were concatenated into a single unified feature vector. This combined vector was then processed through fully connected layers, including dimensionality reduction, non-linear activation (ReLU), dropout (0.3), and a final softmax layer to yield class probabilities for multiclass ovarian tumor classification. This early-fusion (joint projection) approach effectively integrates detailed local feature extraction with global context modeling, leveraging the complementary strengths of CNN and Transformer architectures. A detailed schematic of this strategy is illustrated clearly in Figure 2.

### 2.4 Fusion block (learned early-fusion via joint projection)

Let $f_{\mathrm{CNN}}\in {\mathbb{R}}^{d_{c}}$ and $f_{\mathrm{ViT}}\in {\mathbb{R}}^{d_{t}}$ denote the penultimate features from the CNN and Transformer branches. We form $h_{0}=[f_{\mathrm{CNN}};f_{\mathrm{ViT}}]\in {\mathbb{R}}^{d_{c}+d_{t}}$ and learn a joint projection $z=\phi (W_{1}h_{0}+b_{1})$, followed by dropout, and the classifier $p=\text{softmax}(W_{2}z+b_{2})$.

Here, $W_{1}$ adapts both the relative contribution of each branch and their cross-feature interactions under the multiclass loss, enabling early, end-to-end co-adaptation of local (CNN) and global (ViT) cues. This differs from late fusion (e.g., score averaging or stacking), where interactions are deferred to outputs and gradients cannot shape intermediate features jointly. Regularization (dropout, weight decay) curbs co-adaptation and promotes a compact, data-driven feature subspace.

### 2.5 Training strategy and hyperparameters

Model performance was evaluated using patient-level, stratified 5-fold cross-validation to ensure representative and robust outcomes. Each architecture and hybrid combination was independently trained and assessed in ten separate runs with explicitly set random seeds, facilitating reproducibility. Consistent hyperparameters were applied across all experiments, including a batch size of 32 images and a maximum of 50 training epochs. The Adam optimizer was employed with an initial learning rate of 1e-4, with L2 weight decay, dynamically adjusted through a 0.1-factor reduction after five consecutive epochs without validation loss improvement (ReduceLROnPlateau scheduler). Early stopping based on validation loss was used to halt training once no improvement was observed within a fixed patience window, thereby further reducing the risk of overfitting. The categorical cross-entropy loss function was utilized in all models. Dropout (*p* = 0.3 in the classification head, as detailed in Section 2.3) complemented these measures to provide additional regularization.

### 2.6 Evaluation metrics and statistical analysis

Diagnostic performance was assessed using clinically relevant metrics: accuracy, sensitivity (recall), specificity, area under the ROC curve (AUC-ROC), and area under the Precision-Recall curve (AUC-PR), essential given the significant class imbalance present in the dataset. Complementary metrics, such as the F1-score and Matthews correlation coefficient (MCC), were calculated to ensure a comprehensive performance assessment.

The statistical significance of differences between individual and hybrid models was first assessed through Shapiro–Wilk normality testing. Subsequently, paired Student’s *t*-tests were employed for normally distributed data, while the Wilcoxon signed-rank test was utilized for non-normal distributions. For multiple comparisons, ANOVA with Tukey’s *post-hoc* tests was applied. In all cases, statistical significance was set at *α* = 0.01.

### 2.7 Interpretability and generation of activation maps

To assess the capability of the proposed hybrid CNN–Transformer models in identifying and focusing on clinically relevant regions within ultrasound images, the Grad-CAM technique (Selvaraju et al., 2017) was implemented. Specifically, activation maps were extracted from the last relevant layer before global pooling or before the final classification layer, depending on each architecture.

Grad-CAM was implemented using the publicly available library torchcam.^1^ Preprocessed ultrasound images were propagated through the hybrid models to obtain corresponding activation maps, specifically generated after the adaptive feature fusion block (learned early fusion via joint projection), highlighting areas with the highest diagnostic relevance.

**Figure 3:**
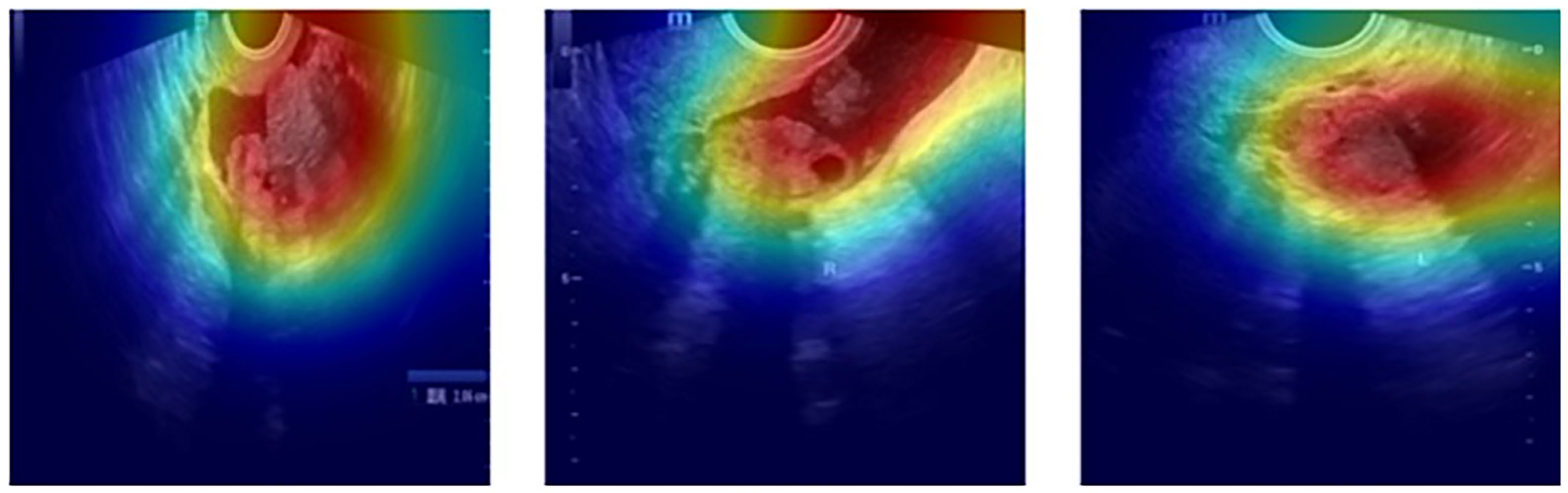
Representative Grad-CAM activation maps of ovarian tumor ultrasound images. Activation heatmaps (red and yellow areas indicating higher activation) generated by the hybrid EfficientNetB7–Swin Transformer model overlaid onto original ultrasound images. These activation maps visually confirm that the model accurately focuses on clinically relevant tumor regions, thus reinforcing the interpretability and diagnostic credibility of the proposed hybrid architecture.

Qualitative evaluation of the generated activation maps was performed by overlaying these maps onto the original ultrasound images using OpenCV and PIL libraries. An activation threshold of 50% of the maximum value was applied to emphasize the regions identified by the models visually. Figure 3 provides representative examples of these Grad-CAM activation maps, clearly illustrating how the hybrid EfficientNetB7–Swin Transformer model effectively focuses on tumor-associated anatomical regions, aligning closely with clinical expertise and expectations.

Visual examination of these maps confirms that model attention is consistently directed toward regions considered clinically significant by experts, reinforcing the interpretability and credibility of the hybrid CNN-Transformer approach in a clinical context. This interpretability facilitates trust in automated diagnostic decisions and represents a critical step towards the practical clinical adoption of artificial intelligence systems.

### 2.8 Software and hardware

The models were implemented and trained using Python version 3.10, employing the PyTorch library version 2.7.0 with CUDA 11.8. Data management and analysis were performed using Pandas 2.1.3, NumPy 1.26.4, and Scikit-learn 1.3.2. All training and studies were conducted on a server equipped with an Intel Xeon Silver 4,216 CPU @ 2.10 GHz and an NVIDIA A100 GPU with 40 GB.

## 3 Results

### 3.1 Comparison of hybrid and individual model performance

**Figure 4:**
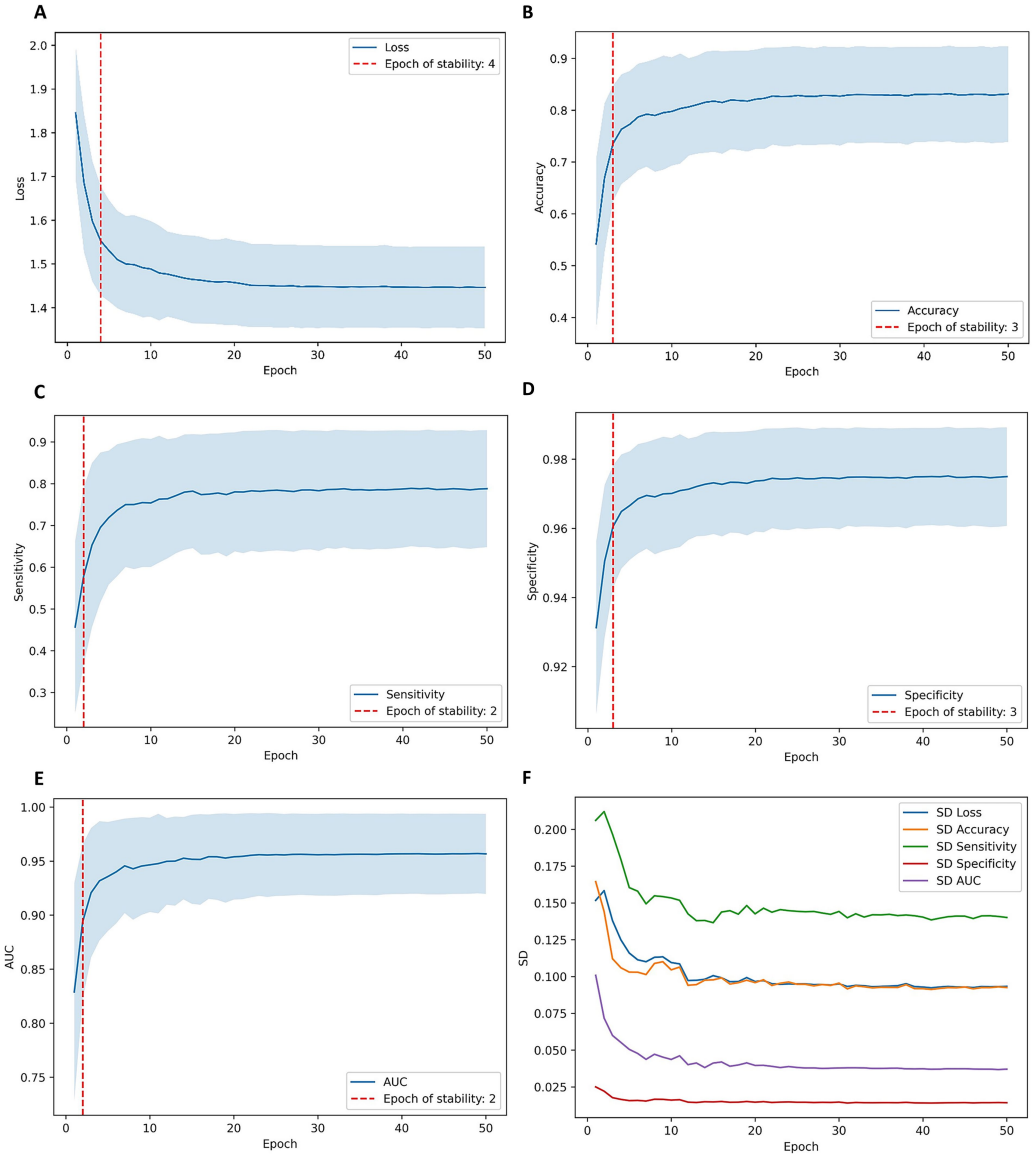
The training stability and variability of the EfficientNet–Swin hybrid model over 50 epochs across ten independent runs. Panels A–E (arranged in two columns and three rows) depict the mean ± standard deviation curves for Loss **(A)**, Accuracy **(B)**, Sensitivity **(C)**, Specificity **(D)** and AUC **(E)**; red dashed lines indicate the epoch of stability for each metric (Loss: 4, Accuracy: 3, Sensitivity: 2, Specificity: 3, AUC: 2). Panel **F** shows the evolution of inter-run variability, plotting the standard deviation across repetitions for all metrics by epoch, which converges as training progresses.

All performance metrics reported in Tables 2–5 correspond to evaluations on the held-out folds of the publicly available OTU-2D dataset (1,469 de-identified B-mode ultrasound images collected at Beijing Shijitan Hospital, Capital Medical University, Beijing, China), using patient-level, stratified 5-fold cross-validation repeated ten times; validation and test partitions were not resampled or augmented to preserve the real-world distribution. The observed, statistically significant improvements of the learned early-fusion models over single-branch baselines across Accuracy, Sensitivity, Specificity, and AUC (Table 2), together with training stability across repetitions (Figure 4), are consistent with the intended benefit of early joint optimization of CNN and Transformer features.

**Table 2:** Bootstrap metrics (*n* = 500) (Accuracy, Sensitivity, Specificity, AUC, F1-macro, MCC) for each algorithm.

Model	Accuracy	Sensitivity	Specificity	AUC	F1	MCC
ResNet152	0.735 [0.707, 0.764]	0.632 [0.590, 0.676]	0.959 [0.955, 0.964]	0.926 [0.913, 0.940]	0.661 [0.619, 0.704]	0.676 [0.641, 0.711]
DenseNet201	0.776 [0.749, 0.802]	0.662 [0.622, 0.700]	0.966 [0.962, 0.970]	0.922 [0.905, 0.938]	0.689 [0.649, 0.727]	0.728 [0.697, 0.758]
EfficientNetB7	0.750 [0.721, 0.779]	0.656 [0.618, 0.698]	0.962 [0.958, 0.967]	0.911 [0.893, 0.929]	0.675 [0.635, 0.716]	0.697 [0.663, 0.731]
VITB16	0.686 [0.653, 0.714]	0.587 [0.547, 0.627]	0.953 [0.948, 0.957]	0.896 [0.877, 0.914]	0.595 [0.553, 0.635]	0.620 [0.580, 0.653]
Swin	0.719 [0.690, 0.747]	0.634 [0.594, 0.673]	0.958 [0.954, 0.962]	0.917 [0.902, 0.933]	0.637 [0.598, 0.672]	0.659 [0.626, 0.692]
ResNet152-VITB16	0.913 [0.898, 0.925]	0.912 [0.898, 0.925]	0.988 [0.986, 0.989]	0.990 [0.987, 0.992]	0.908 [0.893, 0.920]	0.900 [0.883, 0.915]
DenseNet201-VITB16	0.908 [0.893, 0.923]	0.907 [0.894, 0.922]	0.987 [0.985, 0.989]	0.988 [0.984, 0.991]	0.907 [0.893, 0.922]	0.895 [0.879, 0.912]
EfficientNetB7–VITB16	0.905 [0.892, 0.919]	0.906 [0.893, 0.919]	0.986 [0.985, 0.989]	0.985 [0.981, 0.988]	0.904 [0.891, 0.918]	0.892 [0.876, 0.908]
ResNet152–Swin	0.915 [0.901, 0.929]	0.917 [0.903, 0.931]	0.988 [0.986, 0.990]	0.992 [0.989, 0.994]	0.914 [0.900, 0.929]	0.903 [0.887, 0.919]
DenseNet201–Swin	0.913 [0.900, 0.927]	0.915 [0.902, 0.927]	0.988 [0.986, 0.989]	0.990 [0.987, 0.993]	0.913 [0.900, 0.926]	0.901 [0.885, 0.916]
EfficientNetB7–Swin	0.921 [0.907, 0.932]	0.923 [0.910, 0.934]	0.989 [0.987, 0.990]	0.990 [0.987, 0.993]	0.921 [0.908, 0.932]	0.910 [0.894, 0.923]

Values are presented as mean ± 95% CI, with algorithms in rows and metrics in columns.

Metrics are reported as mean [95% CI] on the held-out folds of patient-level, stratified 5-fold cross-validation repeated 10-fold. Validation/test partitions were not resampled or augmented. 95% CIs were computed via bootstrap (*n* = 500). AUC, area under the ROC curve; F1, macro-averaged F1-score; MCC, Matthews correlation coefficient; ViT-B16, Vision Transformer (patch size 16); Swin, Swin Transformer.

**Table 3:** *p*-value results from the statistical analysis (Wilcoxon signed-rank test) comparing EfficientNetB7–Swin with other hybrid models (*n* = 10 measurements).

Model	Accuracy	Sensitivity	Specificity	AUC
ResNet152–VITB16	<0.01	<0.01	<0.01	0.5930
DenseNet201–VITB16	<0.01	<0.01	<0.01	<0.01
EfficientNetB7–VITB16	<0.01	<0.01	<0.01	<0.01
ResNet152–Swin	<0.01	<0.01	<0.01	<0.01
DenseNet201–Swin	0.0488	0.0195	0.0137	0.4922

Wilcoxon signed-rank *p*-values (two-sided) comparing EfficientNet-B7–Swin against each hybrid model across 10 independent repetitions of patient-level, stratified 5-fold cross-validation. Metrics are computed on held-out folds. *α* = 0.01 was used to judge statistical significance. AUC, area under the ROC curve. Note that AUC differences for ResNet152–ViT-B16 and DenseNet201–Swin were not significant (*p* = 0.5930 and *p* = 0.4922, respectively).

**Table 4:** Class-wise metrics (accuracy, sensitivity, specificity, and F1-score), calculated as the average of the ten repetitions of the EfficientNet–Swin hybrid model.

Class (ovarian lesion type)	Accuracy	Sensitivity	Specificity	F1-score
Chocolate cyst	0.8929	0.8238	0.9842	0.8569
Serous cystadenoma	0.8910	0.8859	0.9836	0.8883
Teratoma	0.8969	0.8572	0.9858	0.8766
Theca cell tumor	0.9617	0.9921	0.9943	0.9765
Simple cyst	0.9508	0.9835	0.9937	0.9669
Normal ovary	0.8563	0.8745	0.9802	0.8650
Mucinous cystadenoma	0.9229	0.9731	0.9895	0.9473
High grade serous	0.9914	1.0000	0.9986	0.9957

**Table 5:** Diagnostic performance metrics [accuracy, sensitivity, specificity, AUC, F1-score, and Matthew’s correlation coefficient (MCC)] obtained through soft-ensemble optimization.

Summary statistic	Accuracy	Sensibility	Specificity	AUC	F1	MCC
Mean ± SD	0.933 ± 0.005	0.936 ± 0.003	0.990 ± 0.001	0.991 ± 0.004	0.933 ± 0.005	0.924 ± 0.006

Metrics are presented as mean ± standard deviation (SD), calculated from the combined probabilistic predictions of the top three hybrid CNN–Transformer models (DenseNet201–Swin, EfficientNetB7–Swin, ResNet152–Swin), weighted proportionally by their individual AUC scores.

Table 2 presents a detailed comparison of diagnostic performance between hybrid CNN-Transformer models and individual CNN or Vision Transformer (ViT) architectures. The hybrid EfficientNetB7–Swin Transformer model consistently achieved superior performance, demonstrating an accuracy of 92.13% [95% CI, 90.7–93.2], sensitivity of 92.38% [95% CI, 91.0–93.4], specificity of 98.9% [95% CI, 98.7–99.0], and an area under the ROC curve (AUC) of 0.9904 [95% CI, 0.987–0.993]. Complementary metrics included an F1-score of 0.921 [95% CI, 0.908–0.932] and a Matthews correlation coefficient (MCC) of 0.910 [95% CI, 0.894–0.923].

Before statistical comparisons, normality was assessed using the Shapiro–Wilk test. Depending on normality results, either paired Student’s *t*-tests (for normally distributed differences) or Wilcoxon signed-rank tests (for non-normal distributions) were employed. The EfficientNetB7–Swin hybrid model significantly outperformed individual EfficientNetB7 (accuracy: +17.1 pp., sensitivity: +26.6 pp, specificity: +2.6 pp, AUC: +8.0 pp; *p* < 0.001 for all metrics) and Swin Transformer (accuracy: +20.1 pp, sensitivity: +28.9 pp, specificity: +3.1 pp, AUC: +7.3 pp; *p* < 0.001 for all metrics). Overall, hybrid CNN-Transformer models showed statistically significant superior diagnostic performance across all evaluated metrics compared to individual architectures (*p* < 0.01). Additionally, we conducted a detailed statistical comparison between the EfficientNetB7–Swin model and the other hybrid models using the Wilcoxon signed-rank test. The results are clearly summarized in Table 3.

These statistical findings strongly confirm the significant superiority of the EfficientNetB7–Swin Transformer model across multiple critical metrics. Although some *p*-values for AUC were greater than 0.05 (ResNet152–VITB16 and DenseNet201–Swin), key metrics such as Accuracy, Sensitivity, and Specificity demonstrated statistically significant differences in all cases. This clearly shows the advantage of the EfficientNetB7–Swin model, thus justifying its selection as the optimal model for clinical applications in precise and robust ovarian tumor classification using ultrasound imaging.

Regarding the specific choice of hybrid CNN–Transformer models, it is essential to highlight that the explicit combination between CNN and Transformer architectures was chosen to leverage the complementary strengths of both approaches: the detailed local feature extraction capability of CNNs and the global contextual attention capability of Transformers. While an exhaustive combination of all possible architectures (e.g., ResNet with EfficientNet or ViT-B with Swin Transformer) could potentially explore additional interactions, we chose to focus specifically on CNN-Transformer combinations due to the clear distinction and complementarity of the intrinsic capabilities of these architectures. This decision enabled a more direct and specific comparison and validation of how effectively integrating local and global information significantly enhances medical image classification.

### 3.2 Diagnostic performance by clinical category

**Figure 5:**
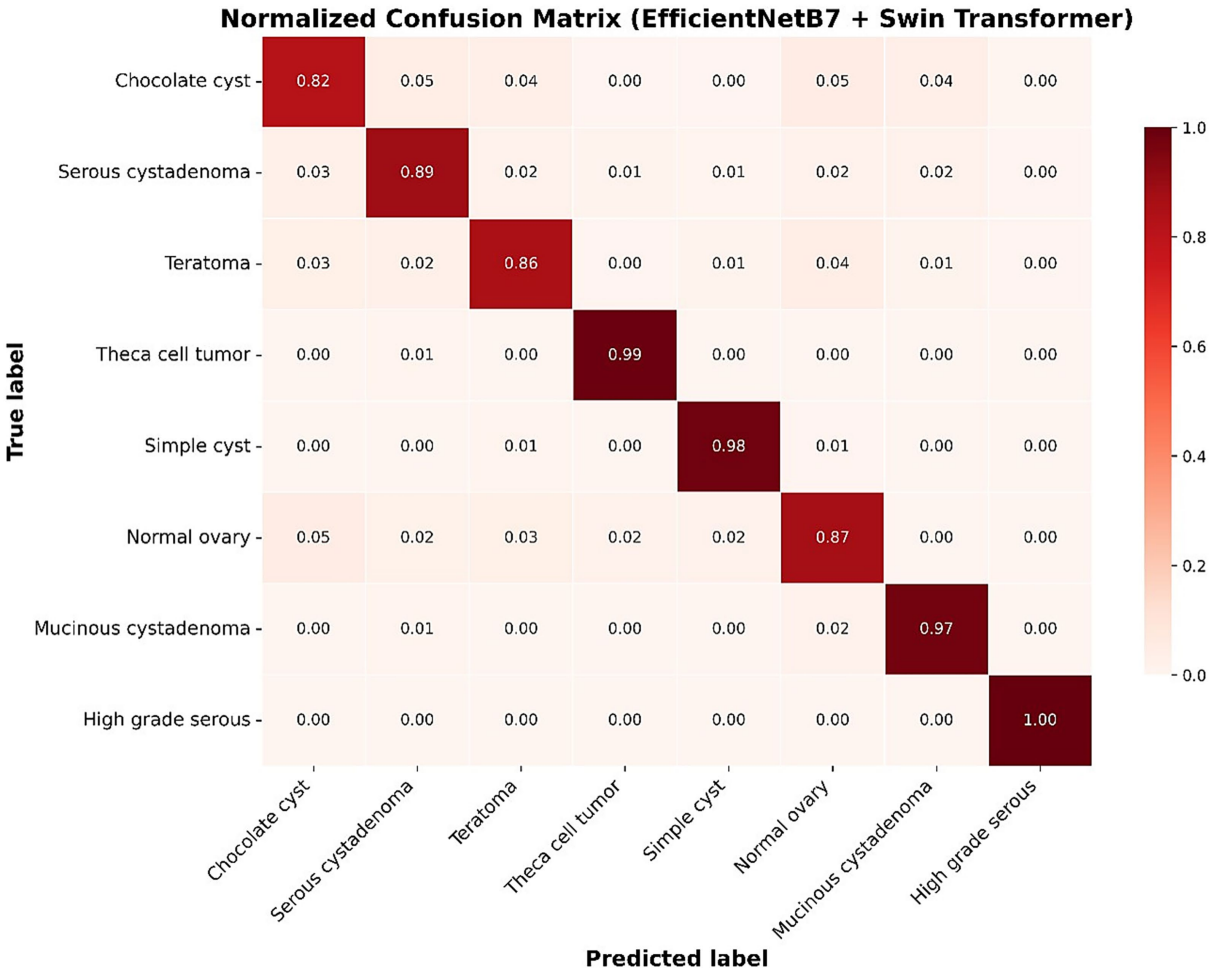
Normalized confusion matrix of the best-performing hybrid EfficientNetB7 + Swin Transformer model.

Figure 5 shows the normalized confusion matrix for the best-performing EfficientNetB7–Swin model. The matrix is strongly diagonal, with class-wise recalls of 1.00 for *high-grade serous carcinoma*, 0.99 for *theca cell tumor*, 0.98 for *simple cyst*, and 0.97 for *mucinous cystadenoma*. Lower—but still robust values are observed for *serous cystadenoma* (0.89), *teratoma* (0.86), *normal o*var*y* (0.87), and *chocolate cyst* (0.82). Most errors are confined to clinically similar cystic entities and occasional confusion with the normal ovary, consistent with overlapping sonographic patterns.

Table 4 provides a detailed breakdown of diagnostic performance by clinical category for the best-performing hybrid EfficientNetB7–Swin model. Categories such as “High-grade serous carcinoma” and “Theca cell tumor” showed outstanding diagnostic performance, with sensitivities approaching 100%, specificities exceeding 99%, and F1-scores above 97%. Similarly, the “Simple cyst” and “Mucinous cystadenoma” categories exhibited high sensitivity (>97%) and specificity (>98%). Even categories characterized by slightly higher error rates, such as “Chocolate cyst” (endometrioma) and “Normal ovary,” retained robust performance (sensitivity ≥82%, specificity ≥98%), indicating strong model generalizability across diverse clinical scenarios.

### 3.3 Training stability and model convergence

Figure 4 illustrates the convergence and stability of diagnostic metrics (loss, accuracy, sensitivity, specificity, AUC) across training epochs for the EfficientNetB7–Swin hybrid model. All performance metrics rapidly converged within ±1 standard deviation of final values early during training (loss stabilized at epoch 4, accuracy at epoch 3, sensitivity at epoch 2, specificity at epoch 3, and AUC at epoch 2). Inter-run variability consistently decreased across epochs, confirming the robustness and reproducibility of the proposed model training strategy.

### 3.4 Probabilistic calibration and clinical decision utility

**Figure 6:**
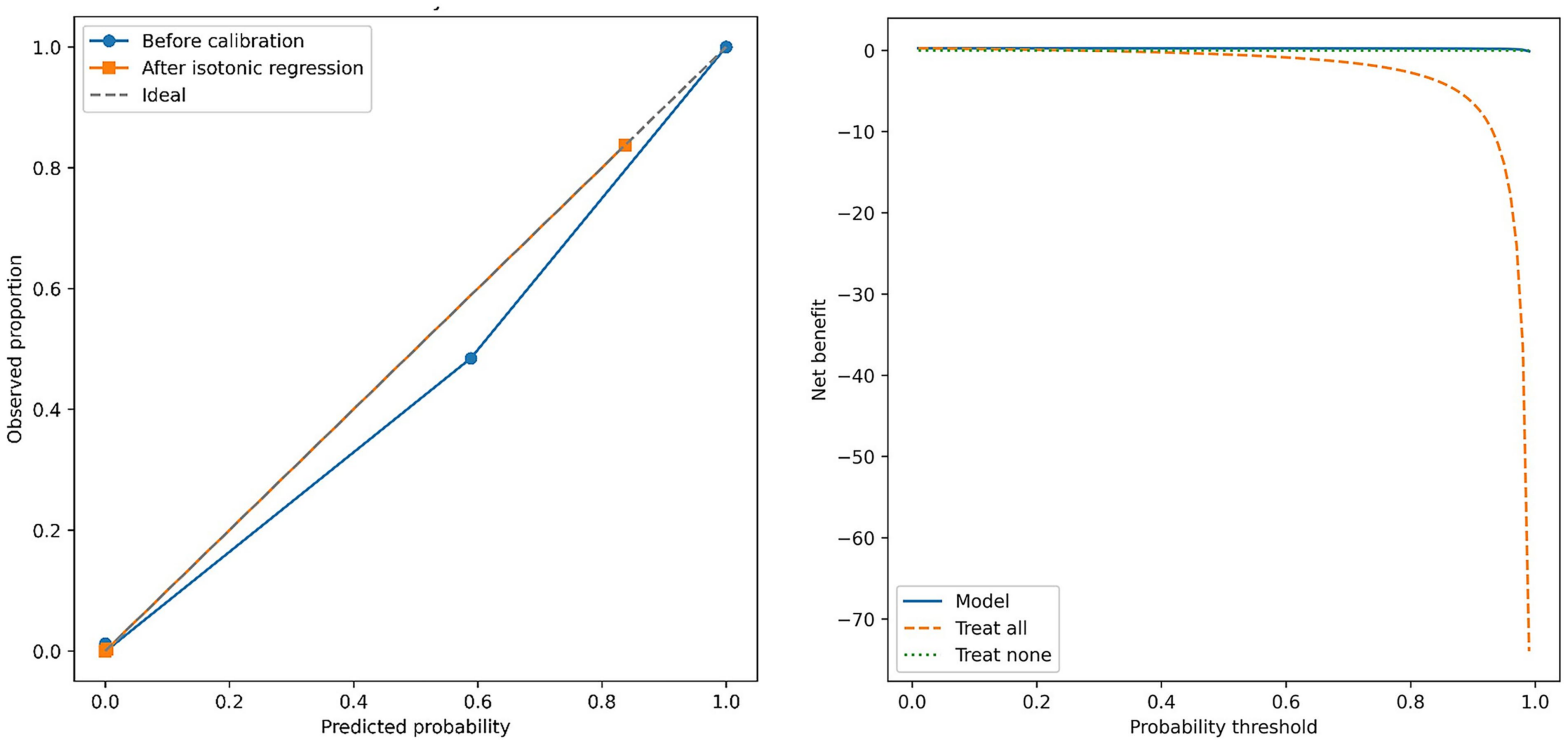
Reliability and Decision Curve Analysis of the EfficientNetB7–Swin hybrid model. Left panel: reliability plot showing the alignment between predicted probabilities and observed outcomes before (blue line with circles) and after isotonic regression calibration (orange line with squares), compared against the ideal calibration reference (dashed gray line). Right panel: Decision curve analysis illustrating net clinical benefit across a range of clinically relevant probability thresholds. The EfficientNetB7–Swin model (solid blue line) demonstrates superior clinical utility compared to the strategies of “treat all” (orange dashed line) or “treat none” (green dotted line).

Figure 6 shows the reliability curves assessing probabilistic calibration for the EfficientNetB7–Swin hybrid model. Initial evaluation revealed a minor underestimation of predicted probabilities, especially at intermediate risk ranges. After isotonic recalibration, predicted probabilities closely matched observed malignancy rates, indicating excellent model calibration.

Clinical decision-curve analysis demonstrated substantial net clinical benefit for the hybrid model compared to standard management strategies (“treat all” or “treat none”) across a clinically relevant threshold range (5–20%). This underscores the practical utility of the model predictions, potentially reducing unnecessary invasive procedures while maintaining high diagnostic accuracy.

### 3.5 Diagnostic performance optimization via soft ensemble

To further enhance diagnostic accuracy, a soft-ensemble approach was employed, combining prediction probabilities from the three highest-performing hybrid models (DenseNet201–Swin, EfficientNetB7–Swin, ResNet152–Swin), weighted proportionally by their individual AUC scores. This strategy yielded a statistically significant additional improvement in overall diagnostic performance, reaching accuracy of 93.3% ± 0.5%, sensitivity of 93.6% ± 0.3%, specificity of 99.0% ± 0.1%, and AUC of 0.991 ± 0.004, along with increased stability in predictions.

### 3.6 Predictive uncertainty and confidence-error analysis

**Figure 7:**
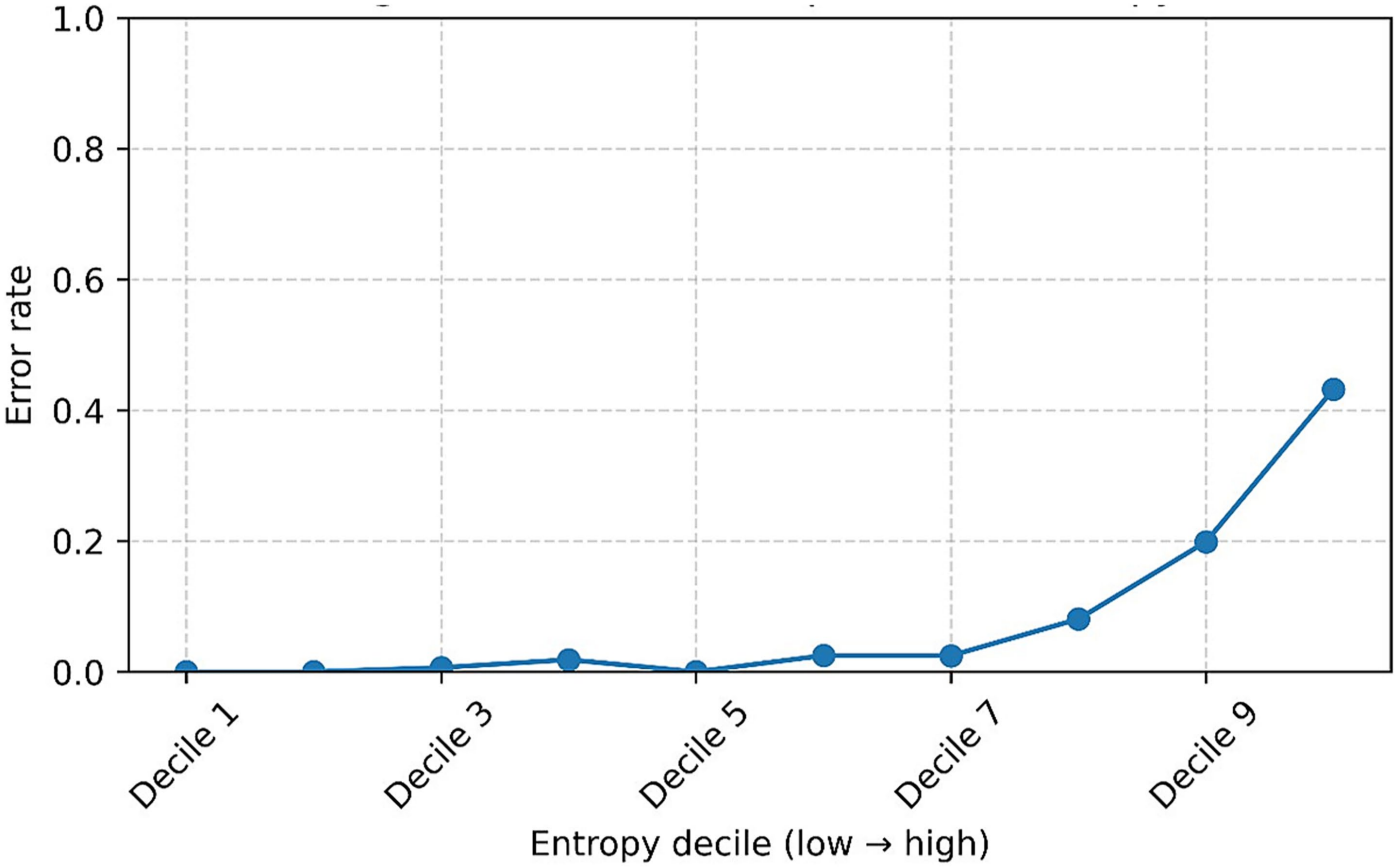
Error rate as a function of prediction entropy, grouped by deciles (1 = lowest entropy/highest confidence → 10 = highest entropy/lowest confidence). The curve clearly illustrates an increase in error rate as prediction uncertainty rises, underscoring the practical utility of implementing entropy-based criteria to guide clinical decision-making.

Figure 7 explores the relationship between predictive uncertainty, measured by entropy of model predictions, and diagnostic error rates. Predictions exhibiting high confidence (low entropy, deciles 1–5) demonstrated negligible error rates. Errors slightly increased from decile 7 (~2%), becoming notably elevated in the highest uncertainty deciles, reaching approximately 8% in decile 9 and 43% in decile 10. These results indicate that entropy is a reliable marker of uncertainty, supporting a practical clinical strategy to automate decision-making for approximately 90% of cases characterized by low uncertainty, while reserving expert clinician review for the remaining 10% exhibiting higher uncertainty. This approach would significantly optimize diagnostic accuracy and efficiency.

## 4 Discussion

### 4.1 Early-fusion hybrid performance

Recent AI-driven ultrasound studies for ovarian tumor classification have predominantly focused on benign–malignant binary discrimination, often assessed only on internal cohorts and lacking external validation (Giourga et al., 2024). For example, Gao et al. (2022) developed a deep learning model with strong multicenter performance on a large dataset but restricted the task to binary detection (Gao et al., 2022). Similarly, a recent pipeline by Dai et al. (2024) achieved robust performance with multiple external validations, yet remained limited to benign–malignant discrimination (Dai et al., 2024). Truly multiclass approaches (differentiating multiple histological subtypes) remain uncommon (Wu et al., 2018). One exception is the CNN-based study by Wu et al. (2023), which addressed seven ovarian tumor subcategories and reported high internal accuracy (Wu et al., 2018); however, it did not include model calibration, uncertainty analysis, or external testing. Most prior work also relies on CNNs alone and omits modern interpretability or clinical-utility analyses. By contrast, we present an early-fusion hybrid CNN–Transformer with a concise, clinically oriented evaluation. We summarize once: isotonic calibration, entropy-based uncertainty, decision-curve analysis, and Grad-CAM; all metrics are reported with 95% confidence intervals.

In our experiments, the EfficientNet-B7–Swin early-fusion hybrid outperformed single-architecture baselines and other hybrids, achieving an accuracy of 92.13%, a sensitivity of 92.38%, a specificity of 98.9%, and an AUC of 0.9904. A soft ensemble of the top hybrids further improved performance to an accuracy of 93.3%, sensitivity of 93.6%, specificity of 99.0%, and AUC of 0.991, underscoring robustness and potential clinical utility. Table 6 summarizes the soft-ensemble’s diagnostic performance (accuracy, sensitivity, specificity, AUC, F1-score, MCC), enabling direct comparison with prior ultrasound AI studies of ovarian tumors.

**Table 6:** Comparison of recent AI-based ultrasound studies for ovarian tumor classification.

Study	Cohort	Modality/task	Number of classes	Model	Validation	External validation	Primary metric (test set)	Explainability/calibration/DCA	Key notes
This work	1,469 images; single-center (China)	2D US (B-mode); multiclass classification	8 (histological subtypes)	Hybrid EfficientNet-B7 + Swin Transformer (early fusion).	5-fold CV (patient-level), 10 × repetition; oversampling; no leakage.	No	AUC 0.9904; Acc 92.13%; Sens 92.38%; Spec 98.90%; ensemble AUC 0.991 (±95% CI)	Yes (Grad-CAM)/Yes (isotonic)/Yes (DCA)	Uncertainty analysis performed; metrics reported with 95% CIs
Gao et al. (2022)	~105,000 US images (3,755 malignant vs. ~ 101 k benign); multi-center (China)	2D US (B-mode); binary classification (benign vs. malignant)	2 (benign vs. malignant)	Deep CNN (custom DCNN model)	Hold-out split (training + internal test); patient-level separation	Yes—two external test sets (multi-center, China)	AUC 0.911 (95% CI 0.886–0.936) internal; external AUC 0.870 & 0.831; Acc ~ 88.8%; Sens ~82.7%; Spec ~88.7%	No/No/No	DCNN outperformed average radiologists and improved non-expert sensitivity on a large, histopathology-confirmed retrospective dataset.
Dai et al. (2024)	~8,500 US images from 21 hospitals (train + internal test); plus external sets: 1,896 images + 159 videos from 2 hospitals (China)	2D US (B-mode) with ROI segmentation; binary classification	2 (benign vs. malignant)	Multi-task CNN (Ovarian Multi-Task Attention Network—OvaMTA: ovary detection + tumor classifier)	Hold-out (internal test cohort held out from training); patient-level split	Yes—external image and video test sets (2 hospitals)	AUC 0.941 (internal image test); AUC 0.941 (external image test); video test AUC 0.911; e.g. video Acc 86.2%, Sens 81.8%, Spec 89.2%	Partial (model provides heatmaps)/No/No	Automated ovary/tumor detection and classification; performance matched senior radiologists; AI assistance improved junior doctors’ accuracy.
Wu et al. (2018)	328 patients (1,142 US images); single-center (China)	2D US (B-mode); multiclass classification (histologic tumor types)	7 (6 benign subtypes + 1 malignant)	Transfer learning with multiple CNNs (VGG16, GoogleNet, ResNet34, ResNeXt50, DenseNet121/201); best model: ResNeXt50	Hold-out split (training/validation/test sets); patient-level separation	No	Acc 95.2%; Sens ≥90%; Spec ≥95% for most classes (HGS carcinoma Sens 90%, Spec 99.2%); overall high AUC (~0.95)	Yes (CNN heatmaps)/No/No	ROI segmentation improved accuracy; addressed multiple subtypes; small dataset; no external validation.
Giourga et al. (2024)	585 patients (3,510 images: 390 benign, 195 malignant); single center (Greece)	2D US (B-mode); binary classification	2 (benign vs. malignant)	Ensemble of three pre-trained CNNs (VGG16, ResNet50, InceptionV3) with optimized weighting	5-fold cross-validation (80/20 splits); patient-level; no single hold-out test	No	AUC ~ 0.922 (95% CI ~ 0.908–0.971); Acc 90.9%; Sens 96.5%; Spec 88.1% (at optimized threshold 0.2)	No (misclassifications reviewed only)/No/No	Sensitivity-optimized threshold (0.2); tuned ensemble (VGG16 50%); sensitivity comparable to expert sonographers.
Xie et al. (2024)	519 patients (269 benign, 250 malignant) from 3 centers; O-RADS 4 lesions focus (China)	2D US (B-mode); binary classification (indeterminate-risk masses)	2 (benign vs. malignant)	Two-stage DeepLabV3 + YOLOv8 pipeline (lesion segmentation + concept-based classification)	Hold-out (train/val/test = 426/46/47 patients); patient-level split; multi-center internal data	No (multicenter data split internally)	AUC 0.950 (95% CI 0.91–0.96); Acc 94.1%; Sens 92.5%; Spec 95.5% (on test set)	Partial (feature concepts via YOLO)/No/No	O-RADS 4 focus; CNN-segmented ROI; strong internal performance; no independent external validation.
Du et al. (2024)	849 patients (train/test 8:2 split); single center (China)	2D US (B-mode); multiclass classification (benign, borderline, malignant)	3 (benign, borderline, malignant)	Hybrid radiomics + deep CNN + clinical features (“DLR” signature combining handcrafted features, transfer-learned CNN output, and clinical data)	Hold-out (random 80/20 split into train and test); patient-level separation	No	AUC (micro-average) 0.90; macro-AUC 0.84 on test; class-specific AUC ~ 0.84 for each category. Borderline tumor detection was poorest (e.g., ~55% recall)	No / No / No	Logistic-regression nomogram fusing radiomics and CNN; first US 3-class (incl. borderline); improved accuracy over either alone; no external validation.
Du et al. (2024)	849 patients (all tumors, task 1) + 391 patients (O-RADS 4–5 subset, task 2); single-center (China)	2D US (B-mode); binary classification (malignant risk prediction)	2 (benign vs. malignant)	Combined deep CNN + radiomics + clinical model (logistic regression nomogram integrating CNN, radiomic, and O-RADS features)	Hold-out (8:2 split for each task); patient-level; two separate analyses (general cohort and high-risk subset)	No	AUC 0.928 (test, all-tumor cohort); AUC 0.869 (test, O-RADS4&5 subset); well-calibrated (Hosmer–Lemeshow *p* > 0.05); DCA showed positive net benefit	Yes (Grad-CAM)/Yes (calibration curves)/Yes (decision curve)	DLR_Nomogram matched expert O-RADS; included calibration and DCA; supports AI parity with standardized risk models.
Barcroft et al. (2024)	577 adnexal masses (1,444 images)—development (UK); 184 masses (476 images)—external test (Italy)	2D transvaginal US; binary classification	2 (benign vs. malignant)	End-to-end CNN + radiomics model (U-Net segmentation + ML classifier using radiomic features from ROI)	Split into training & validation (UK dataset) and independent test (Italian dataset); patient-level	Yes—external test on an independent Italian dataset	AUC 0.90 (external test); F1-score 0.83 on external; Segmentation Dice ≈0.85; achieved sensitivity ~83% on external (comparable to expert assessment)	No/No/No	Automated segmentation standardized ROIs; AUC ≈ 0.90 matched expert sonographers and IOTA models, supporting multicenter generalizability.

US, ultrasound; AUC, area under the ROC curve; 95% CI, 95% confidence interval; Acc, accuracy; Sens, sensitivity; Spec, specificity; CV, cross-validation; ROI, region of interest; DCA, decision-curve analysis; CNN, convolutional neural network; ViT, Vision Transformer; Grad-CAM, Gradient-weighted Class Activation Mapping; O-RADS, Ovarian-Adnexal Reporting and Data System; IOTA, International Ovarian Tumor Analysis.

One key methodological strength of this study lies in the effective management of class imbalance, a prevalent issue in medical imaging datasets. The train-only, patient-level random oversampling strategy, combined with ultrasound-specific augmentation and regularization, improved performance across diagnostic categories, particularly in underrepresented classes such as high-grade serous carcinoma and theca cell tumors, while preserving stable convergence and interpretability.

Several limitations warrant mention. First, this study relies on a single-center dataset (OTU-2D), which may constrain generalizability across populations and imaging settings; multicenter external validation in diverse cohorts is needed. Second, although the proposed early-fusion hybrid outperformed single backbones, additional CNN-Transformer combinations could be explored to assess incremental gains. Finally, we employed train-only oversampling with ultrasound-aware augmentation and strong regularization, but intentionally omitted Mixup/SMOTE/generative augmentation in the primary analysis to avoid synthetic-data artifacts. Future work will benchmark these techniques under identical patient-level splits and multicenter validation to quantify their added value.

Future directions should emphasize external validation and further methodological refinement, including the evaluation of other CNN–Transformer combinations and the expansion of our hybrid framework to additional medical-imaging contexts. Such efforts will reinforce generalizability and robustness, ultimately enhancing clinical adoption and patient outcomes (Eralp and Sefer, 2024; Sefer, 2025).

### 4.2 Clinical and methodological significance

Our fusion operator is a learned early-fusion joint projection; we do not claim a new theoretical fusion family. Its role is to adaptively re-weight and couple CNN/ViT representations at an early stage, allowing gradients to shape both branches under the multiclass objective. We therefore frame our contribution as a clinically aligned, domain-specific instantiation (multiclass ovarian US with calibration, DCA, and uncertainty), rather than a new fusion theory; head-to-head early- vs. late-fusion benchmarking is outlined as future work under identical splits.

For clinical use, decision support must deliver reliable, actionable, and auditable outputs. Well-calibrated probabilities enable threshold-based triage and shared decision-making. Decision-curve analysis quantifies the net benefit versus standard strategies across relevant thresholds. Uncertainty estimates define safe automation boundaries and escalation pathways. Attribution maps (e.g., Grad-CAM) support case-level quality assurance and error analysis, fostering clinician trust.

Methodologically, the framework prioritizes deployment readiness, encompassing bias-aware training (train-only resampling coupled with ultrasound-specific augmentation and regularization), reproducible evaluation with confidence intervals, and transparent reporting that facilitates monitoring, auditing, and future regulatory submissions.

While many hybrid CNN-Transformer designs exist, most target binary tasks or other modalities and are not directly comparable without substantial re-implementation under identical ovarian-US conditions. Our contribution is a domain-specific early-fusion instantiation tuned to ultrasound signal characteristics and class imbalance, embedded in a clinically aligned evaluation and governance template. Future work will focus on multicenter external validation and standardized head-to-head comparisons to confirm generalizability and establish adoption thresholds.

### 4.3 Backbone selection and scope

We intentionally used established CNN and ViT backbones to provide a stable, reproducible reference while foregrounding our learned early-fusion (joint projection) architecture and clinically oriented evaluation (patient-level repeated cross-validation with 95% CIs, isotonic calibration, decision-curve analysis, and entropy-based uncertainty). Given the high performance already observed on this dataset, further replacing backbones with newer SOTA variants may offer limited incremental gains relative to added complexity for clinical deployment. Future work will benchmark more recent families (e.g., ConvNeXt/ConvNeXt-V2, EfficientNet-V2, CoAtNet, MaxViT, Swin-V2) under identical splits and external multicenter validation to assess incremental value.

We did not exhaustively evaluate the latest SOTA backbones; our focus was on a calibrated, uncertainty-aware early-fusion pipeline for eight-class ovarian ultrasound, to be extended with multicenter external validation and targeted SOTA benchmarking in future work.

### 4.4 Regulatory pathways and compliance for clinical integration

Although the present work is a research-grade model, translating an AI-enabled ultrasound classifier into clinical use entails conformity with medical-device and AI governance frameworks. In the EU, our software would qualify as Medical Device Software (MDSW) under MDR 2017/745 (Rule 11), with likely Class IIa/IIb classification depending on intended use and risk; CE-marking would require an ISO-13485 quality management system, risk management per ISO 14971, a software life-cycle process per IEC 62304, human-factors/usability engineering per IEC 62366–1, and a clinical evaluation aligned with IMDRF SaMD guidance (Medical Device Coordination Group (MDCG), 2025; International Organization for Standardization, 2025).

The EU AI Act imposes further obligations on high-risk health AI systems (e.g., risk management, data governance, logging, transparency, and human oversight) with staged timelines. Our deployment plan anticipates an AI Act-aligned technical file and post-market monitoring (Wellnhofer, 2022).

In the United States, this software would be regulated as SaMD by the FDA (510(k) or *De Novo*, depending on predicates). We align development and evaluation with the joint FDA–Health Canada–MHRA principles for Good Machine Learning Practice (GMLP) and transparency, and we would include a Predetermined Change Control Plan (PCCP) in the marketing submission to enable controlled, auditable model updates. Cybersecurity requirements are addressed per the FDA’s premarket cybersecurity guidance (including SBOM, threat modeling, and vulnerability management) (U.S. Food and Drug Administration, Health Canada & Medicines and Healthcare Products Regulatory Agency, 2025).

Operationally, safe clinical integration requires human-in-the-loop oversight, clear intended-use labeling, and auditable interoperability with PACS/EHR systems (DICOM/DICOM-SR, DICOMweb, HL7-FHIR). Our deployment plan includes calibration monitoring, drift detection, and real-world performance surveillance, which are documented within an AI governance program (e.g., NIST AI Risk Management Framework) and a post-market change control process. Finally, we anticipate site-level privacy and security controls (pseudonymisation, encryption, role-based access, audit logs), local DPIAs where required, and jurisdiction-specific data-transfer mechanisms (e.g., SCCs) or data-residency constraints (e.g., PIPL).

These compliance and workflow provisions complement our technical results, outlining a regulatory-ready path from research to deployment that prioritizes patient privacy, safety, and accountability. By specifying device classification, change control, security, and data-protection safeguards, we clarify the steps required for real-world adoption.

### 4.5 Key research gaps in AI-based ovarian ultrasound classification

Most deep learning models for ovarian ultrasound have been evaluated only on single-center cohorts, which limits the evidence of their robustness across different scanners, patient demographics, and clinical settings. This lack of external validation raises concerns about the generalizability of the findings to broader populations and diverse imaging conditions. Current studies have not yet met the stringent requirements for clinical translation, as no AI ultrasound classifier for ovarian tumors has undergone prospective, multi-site trials or demonstrated compliance with medical device regulations. This gap in regulatory readiness underscores the need for further prospective evaluations to ensure safety, efficacy, and seamless integration into real-world workflows.

Present algorithms struggle with underrepresented histological subtypes (e.g., borderline ovarian tumors), which often yield suboptimal detection rates due to scarce training examples. For instance, a recent multiclass model showed markedly low recall (~55%) for borderline tumors (Du et al., 2024), underscoring the need for methods that can recognize rare or intermediate malignancies with higher reliability. Few studies rigorously assess whether model output probabilities reflect actual risk or evaluate clinical net benefit. Most published models report accuracy metrics without calibrating predictions or performing decision-curve analysis, thereby failing to quantify clinical utility in terms of the number of avoided interventions versus the number of missed cancers. Incorporating these analyses is crucial to determine if AI models would improve patient outcomes over standard care.

The prevailing literature seldom quantifies predictive uncertainty, leaving clinicians blind to the confidence of a model’s verdict. Without uncertainty measures, current systems cannot distinguish between cases suitable for automated AI diagnosis and those that require expert review, hindering safe implementation. Notably, prior studies have often omitted any uncertainty analysis, highlighting a need for frameworks that flag low-confidence predictions to inform risk-aware clinical decision-making. There is no consensus on how to define and extract the tumor region in ultrasound images across studies. Variability in ROI selection (manual cropping vs. automatic segmentation) introduces inconsistencies that hinder reproducibility. Automated, standardized ROI segmentation has been shown to improve performance and generalizability (achieving AUC ≈ 0.90, comparable to expert assessments). Yet, most studies have not adopted uniform ROI protocols, representing a critical methodological gap.

The potential of hybrid architectures that combine convolutional neural networks with Vision Transformers remains untapped, mainly in ovarian ultrasound research. Only a handful of studies have examined such hybrids for ovarian tumor classification, so their purported advantages over traditional CNN-based or radiomics models are not fully established. A systematic benchmarking of early-fusion CNN-Transformer models is needed to verify performance gains and guide best practices for leveraging both local and global image features.

## 5 Conclusion

In conclusion, this study presents a rigorously validated hybrid CNN–Transformer model, specifically designed for early fusion (joint projection) multiclass ovarian tumor classification using ultrasound imaging. The EfficientNetB7–Swin Transformer combination notably demonstrated superior diagnostic performance over conventional CNN or Transformer architectures, achieving high accuracy, sensitivity, specificity, and robust calibration.

The use of soft-ensemble methods further enhanced diagnostic precision, illustrating the value of integrating multiple top-performing models. Importantly, visual interpretability provided by Grad-CAM confirmed the clinical relevance of model predictions, significantly improving clinical trust and facilitating adoption into routine diagnostic workflows. Predictive uncertainty analysis using entropy further optimized clinical efficiency by distinguishing cases requiring expert review from those suitable for automated assessment.

The clinical adoption of this model could substantially transform current ultrasound diagnostic protocols for ovarian tumors by significantly increasing diagnostic precision and reducing unnecessary invasive interventions. By integrating automated, interpretable decision-support into routine clinical practice, healthcare providers can streamline diagnostic workflows, prioritize critical cases for expert review, and allocate medical resources more efficiently, thereby directly improving patient outcomes and the overall effectiveness of ovarian cancer care.

Future research efforts must focus on external multicenter validations and further methodological exploration of additional CNN-Transformer combinations to reinforce the practical applicability and generalizability of the proposed hybrid approach. Overall, this study represents a significant step toward enhancing the precision, reliability, and interpretability of AI-driven ovarian tumor diagnostics, which may lead to earlier and more accurate clinical diagnoses, improved patient outcomes, and more efficient clinical management.

## Data Availability

The original contributions presented in the study are included in the article/supplementary material, further inquiries can be directed to the corresponding author.

## Footnotes

^1^ https://github.com/frgfm/torch-cam
